# Association of HLA-DP/DQ and STAT4 Polymorphisms with HBV Infection Outcomes and a Mini Meta-Analysis

**DOI:** 10.1371/journal.pone.0111677

**Published:** 2014-11-03

**Authors:** Yun Liao, Bei Cai, Yi Li, Jie Chen, Chuanmin Tao, Hengjian Huang, Lanlan Wang

**Affiliations:** Department of Laboratory Medicine, West China Hospital, Sichuan University, Chengdu, Sichuan Province, China; University of Pisa, Italy

## Abstract

**Background:**

Though HLA-DP/DQ is regarded to associate with HBV susceptibility and HBV natural clearance, its role in hepatocellular carcinoma (HCC) development is obscure. And the role of STAT4 in HBV susceptibility and clearance as well as HCC development is still contentious. Therefore, we conducted this study, aiming to clarify these obscure relationships.

**Methods:**

We recruited 1312 Chinese Han subjects including healthy controls, HBV carriers and HCC patients in the experiment stage. The meta-analysis included 3467 HCC patients and 5821 HBV carriers to appraise the association with HCC development.

**Results:**

Consistent with previous studies, HLA-DP/DQ associated with HBV susceptibility and HBV natural clearance (p<0.05). However, the experiment showed that HLA-DP rs3077, rs9277535 and rs7453920 did not associate with HCC development (dominant model, rs3077, OR = 0.86, 95%CI = 0.62–1.18; rs9277535, OR = 0.94, 95%CI = 0.68–1.30; rs7453920, OR = 0.75, 95%CI = 0.44–1.27). Meta-analysis again consolidated this conclusion (allele model, rs3077, OR = 0.94, 95%CI = 0.87–1.02; rs9277535, OR = 1.04, 95%CI = 0.97–1.11; rs7453920, OR = 0.89, 95%CI = 0.76–1.02). As for STAT4 rs7574865, we did not find any significant association with HBV susceptibility (OR = 0.91, 95%CI = 0.66–1.26) or HBV natural clearance (OR = 1.13, 95%CI = 0.86–1.49). Moreover, current data failed to acquire positive connection of rs7574865 with HCC development (experiment, OR = 0.86, 95%CI = 0.62–1.19; meta-analysis, OR = 0.87, 95%CI = 0.74–1.03), which may be due to the small sample size.

**Conclusions:**

HLA-DP/DQ polymorphisms (rs3077, rs9277535, rs7453920) did not associate with HCC development, but did correlate with HBV susceptibility and HBV natural clearance. STAT4 rs7574865 seemed not to correlate with HBV susceptibility or natural clearance. And it seemed rather ambiguous in its role on HCC development at present.

## Introduction

Hepatitis B virus (HBV) infection is one of the major causes of chronic hepatitis and the main risk factor for liver cirrhosis and hepatocellular carcinoma (HCC) [1]. Approximately 350 to 400 million people are HBV carriers worldwide and 1 million deaths from HBV-related diseases, including cirrhosis, liver failure and HCC [2].

Most HBV infections which occurred in adults are often self-limited, with spontaneous clearance of HBV from blood and liver. However, some of the infections in adults do not resolve but develop into persistent infection (though less than 5%) [3]. After HBV persistent infection, about 20% patients would progress to cirrhosis and 5–10% would develop HCC. As for the reason why some adults could achieve spontaneous clearance, and some would develop into chronic hepatitis and even HCC, still remains to be further illustrated. Recent Genome-Wide Association studies (GWAS) revealed that HLA-DP/DQ polymorphisms (HLA-DP rs3077, rs9277535, HLA-DQ rs2856718, rs7453920) associated with HBV clearance [4], [5], from a genetic perspective to investigate the etiology of chronic hepatitis B. Subsequently, these findings were validated by many a study [6]–[10], especially in Asians, like Chinese Han, Japanese, Korean and so on. Hu and his colleagues [7] first pointed out that HLA-DP/DQ not only associated with HBV clearance, but also correlated with HCC development, with HLA-DQ rs2856718 significantly decreasing HCC risk and rs3077 an approaching significant effect on HCC development. Moreover, in their further research [11], they found additional HLA-DQ single nucleotide polymorphism (SNP) rs9272105 correlated with HCC development, however, when they validated the previous SNPs, they only found rs9277535 pertained to HCC development, which was regarded as insignificant in their previous study [7], vice versa, the previous proved-to-be significant SNPs (rs3077, rs2856718) did not reach statistical significance again. Additionally, several other studies including GWAS studies did not acquire that these SNPs correlated with HCC development [8], [11]–[13]. Therefore, up to now, no confirmative conclusion could be drawn on which SNPs of HLA-DP/DQ would play the decisive role in the development of HCC.

Still, one GWAS study [12] described above also revealed Signal Transducer and Activator of Transcription 4 (STAT4) rs7574865 significantly correlated with HBV-related HCC except for HLA-DQ polymorphism. However, when they examined whether this SNP associated with chronic HBV (CHB) infection, they found results little more than a trend of higher frequency for G allele in chronic HBV carriers compared with those who did not carry HBV. Clark et al [14] recruited Vietnamese as the investigated subjects to explore the relationship between STAT4 rs7574865 and HCC development, only to find a trivial role contributing to HCC development (P = 0.047). And Chen’s study [15] did not find any significant association at all (P = 0.57). These seemingly conflicting results sparked our great interest. Whether STAT4 did exert influence on HCC development as well as HBV seroclearance still remains obscure.

Therefore, in this study, we mainly aimed to investigate the association of HLA-DP rs3077, rs9277535, HLA-DQ rs7453920 and STAT4 rs7574865 with HCC development, chronicity of HBV infection in Han population dwelling in West China, both from experiment and meta-analysis perspective to clarify the currently ambiguous role of these SNPs with HCC development, and to validate their correlations with HBV susceptibility and clearance from the experiment.

## Materials and Methods

### Patients

The present study recruited 1312 Chinese Han subjects, including 450 chronic HBV carriers, 227 hepatocellular carcinoma patients and 635 healthy controls, from March to July in 2013 in West China Hospital. Chronic HBV carriers were defined as those who had histories of at least 6 months HBsAg positivity (HBsAg+) as well as no HBsAb (anti-HBs negative) existed in the serum, with no evidence supporting cirrhosis or HCC. The diagnosis of HCC was confirmed by a pathological examination and/or alpha-fetoprotein elevation (>400 ng/ml) plus imaging examination (i.e. magnetic resonance imaging and/or computerized tomography). The controls comprised two groups: 398 subjects with HBV natural clearance (NC subjects) and 237 subjects who were negative for all the serum biomarkers of HBV (HBsAg, anti-HBs, HBeAg, anti-HBe, anti-HBc) (healthy control, HC). Subjects with natural clearance (NC subjects) were those who were negative for HBsAg, but positive for both anti-HBs and anti-HBc. All the control subjects had normal liver functions. And those who were coinfected with hepatitis A, C, D virus or human immunodeficiency virus (HIV) were excluded from this study. All patients consented to sample collection for this study and written informed consent was obtained for each participant. This study was approved by the Institutional Review Board of West China Hospital of Sichuan University. The procedures are in accordance with the Helsinki Declaration.

### Serological testing

Serology markers of HBV including HBsAg, HBeAg, anti-HBs, anti-HBe and anti-HBc were analyzed using Elecsys Modular E170 immunoassay (Roche Diagnostics, GmbH, Mannheim, Germany) or I2000 immunoassay (Abbott co., USA) in accordance with manufacturers’ instruction. Clinical biochemistry analysis on ALT, AST, TB, DB, ALP, ALB and GGT were conducted on Cobas c702 assay with photocolorimetric method (Roche Diagnostic, GmbH, Mannheim, Germany). HBV DNA from 200 µl serum was extracted by NucliSENS easyMAG system (Biomerieux Company, Paris, France), the viral load was measured using Roche Light Cycler 480α (Roche Diagnostics, Basel, Switzerland).

### HLA-DP/DQ and STAT4 polymorphism genotyping

All the four SNPs (HLA-DP rs3077, rs9277535; HLA-DQ rs7453920; STAT4 rs7574865) were genotyped using polymerase chain reaction-high resolution melting (HRM) analysis performed on Light Cycler 480 (Roche Diagnostics, Penzberg, Bavaria, Germany). Genomic DNA kit (Biotake Corporation, Beijing, China) was used to extract the free circulating DNA from the blood sample and the concentration was measured by Nanodrop 2000c spectrophometer (Thermo Scientific, DE). SNP genotyping was performed in a 20 µL reaction system contained 10 µL Roche Master Mix (Roche Applied Science) which comprises FastStart Taq DNA Polymerase and the High Resolution Melting Dye in a reaction buffer, 2.4 µL 25 mM MgCl_2_, 0.2 µL 10 µmol/L Forward Primer and 0.2 µL 10 µmol/L Reverse Primer, 6.2 µL deionized water and finally 1 µL DNA sample as recommended by the manufacturer. The whole genotyping process encompasses four programmes, namely, pre-denaturation, amplification, high resolution melting and cooling. When finished, the results were analyzed by the corresponding Gene Scanning Software v1.2 (Roche Diagnostic) primarily based on the shape of the melting curve. Primers used in this study were shown in Table S1.

### Meta-analysis on association of HLA-DP/DQ and STAT4 with HCC development

**Figure 1:**
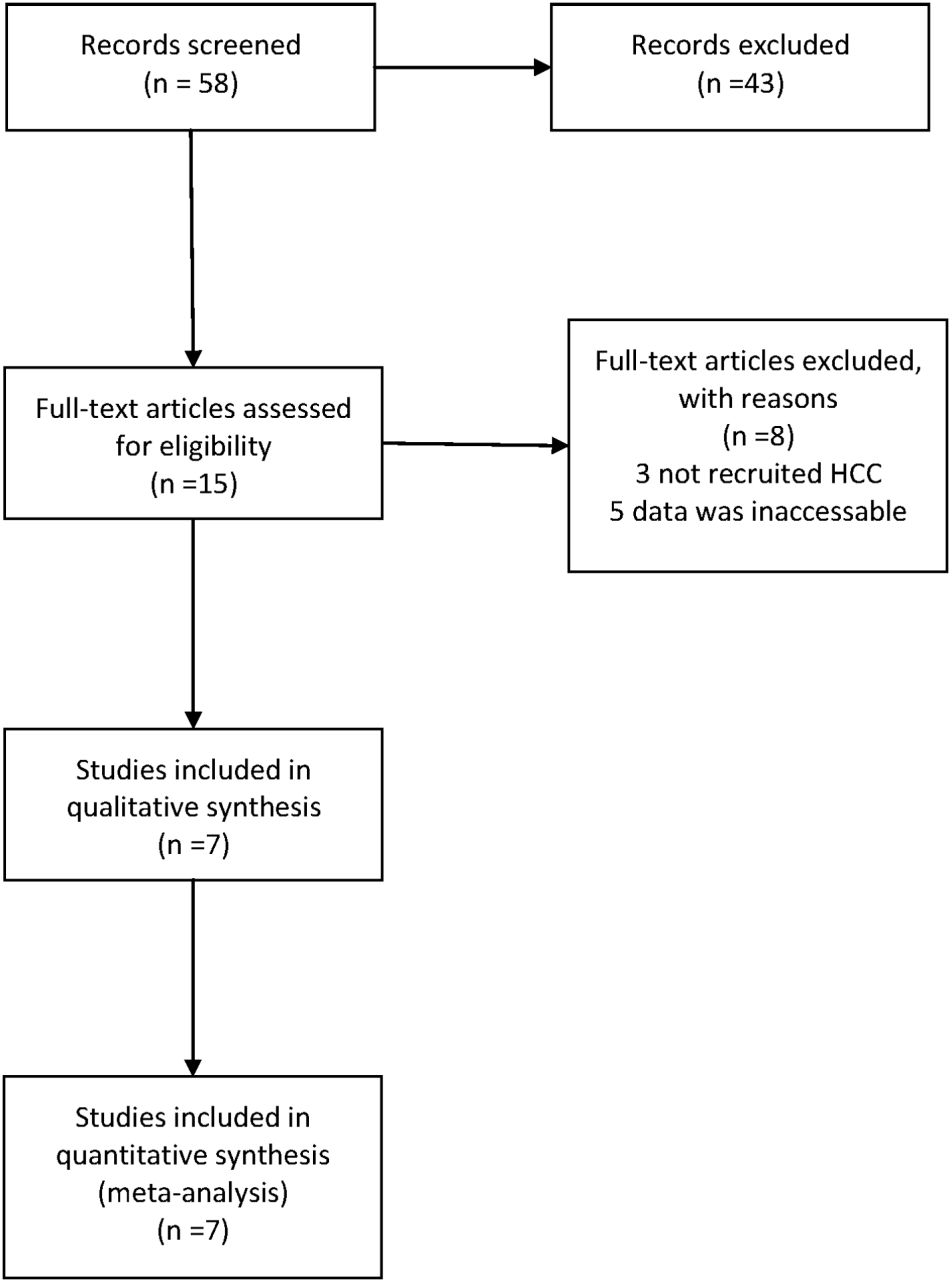
Flow diagram for article screening.

We searched Pubmed, Embase to screen the eligible studies. Such words were used in the search strategy: “HLA-DP” or “HLA-DQ” or “rs3077” or “rs9277535” or “rs7453920” or “STAT4” or “rs7574865” and “liver cancer” or “hepatocellular carcinoma” or “HCC”. The detailed screening process is shown in Figure 1. Data were collected by two investigators (Yun Liao and Bei Cai). The inclusion criteria are listed as follows: 1) the article assessed the genotype frequencies of HLA-DP/DQ or STAT4 between HBV and HCC; 2) study design was a case-control study; 3) the diagnosis of each stage of liver disease conforms to the American Association for the Study of Liver Disease [16], [17]; 4) odds ratio with the 95% confidence interval [95%CI] was reported or could be figured out through the available data. The studies which did not meet above principles were not included. We mainly extracted the odds ratio and the 95%CI from each study so as to fully take advantage of every related study.

Quality of each study included in this study was assessed using the Newcastle-Ottawa Scale (NOS), which has in total 3 categories: 4 stars for selection, up to 2 stars for comparability and 3 stars for exposure/outcome. Nine stars were regarded as the highest quality and studies with more than 6 stars were deemed as the high quality study [18].

### Statistical analysis

Hardy-Weinberg equilibrium (HWE) was independently appraised for each polymorphism. Continuous variables with skewed distribution were described with median and interquartile and the Kruskal-Wallis H test was used for these data. Pearson’s chi-square test or Fisher’s exact test were used to analyze the allele case-control comparisons. Association of SNPs with development as well as susceptibility of HBV infection was estimated by figuring out the odds ratio (OR) and 95% Confidence Interval (CI). When comparing the two group of subjects (case and control), several analytic methods were used: allele frequency distribution of the two groups (allele A versus allele B, A as the major allele, B as the minor allele, this applied to the following methods); genotypic distribution (AA versus AB versus BB); dominant model (AB+BB versus AA). Haplotype analysis was performed to explore whether HLA-DP/DQ polymorphisms were in strong linkage disequilibrium (LD) or they independently contribute to the susceptibility of HBV infection, and can capture additional significant variants since it’s more sensitive than the single SNP analysis. Haplotypes with frequencies of more than 0.03 were analyzed. Pearson’s chi-squared test was used for case–control comparisons.

Meta-analysis was adopted to comprehensively evaluate the correlation of genetic factors with HCC development. The heterogeneity for the included articles was evaluated using Cochran’s Q test, I^2^ statistics (the heterogeneity could be accepted if P>0.1 and I^2^≤50%). If the value is less than 50%, we could think that no heterogeneity existed among the included studies, and then fixed-effects model was used to evaluate the odds ratios. While if I^2^ value is more than 50%, random-effects model was used. Additionally, if all the studies were from one ethnicity, we did not perform subgroup analysis. Sensitivity analysis was performed to evaluate the reliability of the results.

All the analyses were performed using SPSS 13.0 (SPSS Inc., Chicago, IL, USA), STATA 12.0 (version 12.0), comprehensive meta-analysis (version 2.0, Englewood, NJ, USA) and SHEsis online software (http://analysis.bio-x.cn/myAnalysis.php) [19]. A two-sided P value<0.05 was deemed as statistically significant.

## Results

### Clinical characteristics of included subjects

The median age for HC patients was 42 (range: 29–53) years, for NC patients was 49 (range: 41–59) years, for HBV patients was 46 (range: 36–57) years and for HCC patients was 49 (42–59) years. HCC group has a higher proportion of male patients (80.2%), in comparison with HBV group (63.8%), SC group (51.2%) or NC group (38.0%). All the polymorphisms in the four groups were in Hardy-Weinberg equilibrium (P>0.05). The detailed clinical characteristics of the four groups were shown in Table 1.

**Table 1:** Clinical characteristics of subjects recruited in this study.

Group	HC	NC	HBV	HCC
number of subjects	237	398	450	227
age (years)*	42 (29–53)	49 (41–59)	46 (36–57)	49 (42–59)
Gender (male/female)*	90/147 (38.0)	204/194 (51.2)	287/163 (63.8)	191/47 (80.2)
HBsAg+	0	0	450	227
HBsAb+	0	398	0	0
HBeAg+	0	0	65	56
HBeAb+	0	0	369	183
HBcAb+	0	398	450	221
ALT(IU/L)*	16 (11.5–22.5)	20 (14–26)	24 (17–38)	40 (27–64)
AST(IU/L)*	18 (15–22)	21 (17–25)	25 (20–32)	44 (31–66)
TP(g/L)	69.3 (65.3–73.9)	71.4 (67.9–74.7)	71.8(67.6–75.3)	70.4 (66.2–73.6)
ALB(g/L)	43.8 (40.9–46.3)	45.3 (42.9–47.6)	44.9 (42.0–47.3)	40.9 (37.7–43.8)
TB (µmol/L)	11.1 (8.2–14.75)	11.7 (9.5–15.5)	13.8 (10.5–17.8)	15.1 (11.5–20.3)
GGT(IU/L)*	16 (11–26)	18.0 (12.0–28.5)	21 (13–36)	78.0 (43.7–172.7)
PLT*	168 (126.5–211)	178 (139.75–212.25)	152 (114.5–191.0)	116 (75–169)

*Significant difference existed among all the four groups; HC, healthy control; NC, natural clearance subjects; HBV, hepatitis B virus patients; HCC, hepatocellular carcinoma patients; ALT, alanine aminotransferase; AST: aspartate aminotransferase; ALB, albumin; TB, total bilirubin; TP, total protein; GGT, gamma-glutamyl transpeptidase.

Continuous variables were described as median and interquatile range.

We next analyzed the association of HLA-DP/DQ and STAT4 genotypes with clinical indicators in each group. Data showed that rs3077 genotype AA tended to associate with a higher aspartate amino transferase (AST), especially in HCC patients (P = 0.05). But no other polymorphisms were found to correlate with these indicators (HLA-DQ rs7453920 was excluded due to its too skewed distribution of genotypes) (Table S2).

### Association analysis of HLA-DP/DQ, STAT4 polymorphisms with HBV clearance and susceptibility

To identify genetic variations associated with HBV susceptibility, we first compared genotype frequencies of HLA-DP/DQ, STAT4 between HBV group and NC group respectively. The minor allele frequencies of HLA-DP/DQ in both the two groups (HBV vs. NC) were as follows: rs3077, 0.271 vs. 0.324; rs9277535, 0.276 vs. 0.378; rs7453920, 0.067 vs. 0.116; rs7574865, 0.336 vs. 0.336). After adjusting age and sex to control for potential confounders, we observed that rs3077 non-GG genotype, rs9277535 non-GG genotype and rs7453920 non-GG genotype favored HBV seroclearance respectively (adjusted dominant model, rs3077, OR = 0.70, 95%CI = 0.53–0.93; rs9277535, OR = 0.54, 95%CI = 0.40–0.72; rs7453920, OR = 0.48, 95%CI = 0.33–0.70) (Table 2). Allele model further confirmed that rs3077 allele A, rs9277535 allele A and rs7453920 allele A, all had protective effect on HBV seroclearance (rs3077, OR = 0.77, 95%CI = 0.63–0.96; rs9277535, OR = 0.63, 95%CI = 0.51–0.77; rs7453920, OR = 0.55, 95%CI = 0.39–0.77). But STAT4 rs7574865 seemed not to correlate with HBV clearance (dominant model, OR = 0.91, 95%CI = 0.66–1.26, p = 0.571) (Table 2).

**Table 2:** Association of HLA-DP/DQ and STAT4 polymorphisms with HBV susceptibility, HBV natural clearance and HCC development.

Genotype	HC	percentage	NC	percentage	HBV	percentage	HCC	percentage	HBV vs. HC		HBV vs. NC		HCC vs. HBV	
									OR (95%CI)	P	OR (95%CI)	P	OR (95%CI)	P
rs3077														
GG	100	42.2%	175	44.2%	233	53.3%	128	57.1%	1		1		1	-
AG	115	48.5%	185	46.7%	171	39.1%	78	34.8%	**0.64 (0.46–0.89)**	**0.008**	**0.69 (0.52–0.92)**	**0.012**	0.83 (0.59–1.17)	0.289
AA	22	9.3%	36	9.1%	33	7.6%	18	8.0%	0.64 (0.36–1.16)	0.14	0.69 (0.41–1.15)	0.151	0.99 (0.54–1.83)	0.982
Dominant									**0.64 (0.46–0.88)**	**0.006**	**0.69 (0.53–0.91)**	**0.009**	0.86 (0.62–1.18)	0.35
Adjusted*									**0.60 (0.43–0.84)**	**0.003**	**0.70 (0.53–0.93)**	**0.012**	-	-
G	315	66.5%	535	67.6%	637	72.9%	334	74.6%	1		1		1	-
A	159	33.5%	257	32.4%	237	27.1%	114	25.4%	**0.74 (0.58–0.94)**	**0.013**	**0.77 (0.63–0.96)**	**0.017**	0.92 (0.71–1.19)	0.515
rs9277535														
GG	83	35.6%	136	36.8%	226	51.8%	118	53.4%	1		1		1	-
AG	110	47.2%	188	50.8%	179	41.1%	84	37.5%	**0.60 (0.42–0.84)**	**0.003**	**0.57 (0.43–0.77)**	**<0.001**	0.90 (0.64–1.26)	0.54
AA	40	17.2%	46	12.4%	31	7.1%	19	8.5%	**0.28 (0.17–0.48)**	**<0.001**	**0.41 (0.24–0.67)**	**<0.001**	1.17 (0.64–2.17)	0.608
Dominant									**0.51 (0.37–0.71)**	**<0.001**	**0.54 (0.41–0.72)**	**<0.001**	0.94 (0.68–1.30)	0.705
Adjusted*									**0.51 (0.36–0.72)**	**<0.001**	**0.54 (0.40–0.72)**	**<0.001**	-	-
G	276	59.2%	460	62.2%	631	72.4%	320	72.4%	**1**		**1**		1	-
A	190	40.8%	280	37.8%	241	27.6%	122	27.6%	**0.55 (0.44–0.70)**	**<0.001**	**0.63 (0.51–0.77)**	**<0.001**	0.99 (0.77–1.29)	0.989
rs7453920														
GG	199	84.0%	302	77.2%	391	87.9%	203	90.6%	1		1		1	-
AG	33	13.9%	87	22.3%	48	10.8%	21	9.4%	0.74 (0.46–1.19)	0.213	**0.43 (0.29–0.62)**	**<0.001**	0.84 (0.49–1.45)	0.534
AA	5	2.1%	2	0.5%	6	1.3%	0	0.0%	0.61 (0.18–2.03)	0.416	2.32 (0.46–11.56)	0.292		
Dominant									0.72 (0.46–1.13)	0.156	**0.47 (0.32–0.68)**	**<0.001**	0.75 (0.44–1.27)	0.286
Adjusted*									-	-	**0.48 (0.33–0.70)**	**<0.001**	-	-
G	431	90.9%	691	88.4%	830	93.3%	427	95.3%	1		1		1	-
A	43	9.1%	91	11.6%	60	6.7%	21	4.7%	0.72 (0.48–1.09)	0.121	**0.55 (0.39–0.77)**	**<0.001**	0.68 (0.41–1.13)	0.137
rs7574865														
GG	97	40.9%	181	46.3%	190	43.2%	104	46.8%	1		1		1	-
GT	113	47.7%	157	40.2%	204	46.4%	93	41.5%	0.92 (0.66–1.29)	0.634	1.24 (0.92–1.66)	0.151	0.83 (0.59–1.17)	0.295
TT	27	11.4%	53	13.6%	46	10.5%	25	11.2%	0.87 (0.51–1.48)	0.609	0.83 (0.53–1.29)	0.401	0.99 (0.58–1.71)	0.979
dominant	140		210		250		118		0.91 (0.66–1.26)	0.571	1.13 (0.86–1.49)	0.368	0.86 (0.62–1.19)	0.37
G	307	64.8%	519	66.4%	584	66.4%	301	67.8%	1		1		1	-
T	167	35.2%	263	33.6%	296	33.6%	143	32.2%	0.93 (0.74–1.78)	0.555	1.0 (0.82–1.23)	0.998	0.94 (0.73–1.19)	0.602

*Logistic regression analysis adjusted for age and sex. For those insignificant results in univariate analysis, we did not adjust the results; HC, healthy control; NC, natural clearance; HBV, hepatitis B virus patients; HCC, hepatocellular carcinoma.

Next, we appraised the correlation between HBV and HC subjects. HLA-DP rs3077 and rs9277535 still correlated with HBV susceptibility (adjusted dominant model, rs3077, OR = 0.60, 95%CI = 0.43–0.84; rs9277535, OR = 0.51, 95%CI = 0.36–0.72). But we did not find significant association between rs7453920 and HBV susceptibility this time (OR = 0.74, 95%CI = 0.46–1.18) (Table 2). However, since the minor allele frequency of rs7453920 in HBV carriers resembles HCC patients, we combined the two groups and reappraised the correlation of rs7453920 with HBV susceptibility. Univariate analysis showed that rs7453920 tended to associate with HBV susceptibility in dominant model (OR = 0.66, 95%CI = 0.43–1.01, p = 0.053). Additionally, we made a comparison between the control subjects (NC+HC) and virus-carrying group (HBV+HCC). Results showed that HLA-DP rs3077, rs9277535 and even HLA-DQ rs7453920 were significantly pertinent to HBV susceptibility. However, we still did not find significant association existed between STAT4 rs7574865 and HBV susceptibility (P>0.05).

### Association analysis of HLA-DP/DQ, STAT4 polymorphisms with HCC development and the meta-analysis

To identify the correlation of these genetic factors with HBV progression, we compared the genotype frequency between HCC group and HBV group. The minor allele frequencies in the two groups were shown as follows (HCC vs. HBV): rs3077, 0.254 vs. 0.271; rs9277535, 0.276 vs. 0.276; rs7453920, 0.047 vs. 0.067; rs7574865, 0.322 vs. 0.336). Univariate analysis showed that none of the gene polymorphisms associated with HCC development (dominant model, rs3077, OR = 0.86, 95%CI = 0.62–1.18; rs9277535, OR = 0.94, 95%CI = 0.68–1.30; rs7453920, OR = 0.75, 95%CI = 0.44–1.27; rs7574865, 95%CI = 0.86, 95%CI = 0.62–1.19) (Table 2).

**Figure 2:**
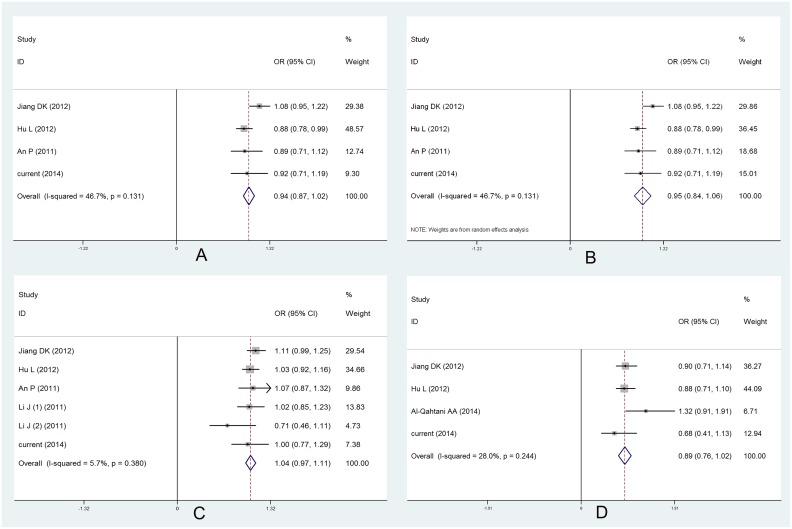
Forest plot for association of rs3077, rs9277535 and rs7453920 with HCC development. (A) Meta-analysis for rs3077 calculated in Fixed-effects model; (B) Meta-analysis for rs3077 calculated in Random-effects model; (C) Forest plot for rs9277535 with HCC development, Li J (1) represents southern Han Chinese, while (2) represents northern Han Chinese; (D) Forest plot for rs7453920 with HCC development.

Then, based on current study, we adopted meta-analysis, from a more powerful way, to illuminate the correlation between these host genetic factors and HBV progression. In total, fifty-eight articles were searched, but only data from seven studies can be extracted, including five studies [7], [8], [12], [20], [21] focused on HLA-DP/DQ, two studies [14], [15] researched STAT4 rs7574865. Quality assessment of all the included studies showed that only study conducted by Al-Qahtani et al. [21] scored less than 6 points and thus was regarded as study of low quality. All the other studies were of high quality. The clinical characteristics as well as the quality scores of the enrolled studies were shown in Table S3 and Table S4. Plus our data, in total, 3467 HCC patients and 5821 HBV carriers were included in this meta-analysis. We extracted the effect size and its 95% confidence interval to appraise the correlation for HLA-DP/DQ and genotype frequencies for STAT4 rs7574865. Meta-analysis based on data from three studies [7], [8], [12] plus our present data, including 2997 HCC patients and 3738 HBV patients, showed that HLA-DP rs3077 did not correlate with HCC development (allele model, OR = 0.94, 95%CI = 0.87–1.02) using fixed model effects (Table 3, Figure 2A). Since moderate heterogeneity existed among all the studies (I^2^ = 46.7%), we adopted random effects model to reappraise the correlation and results also indicated its insignificant association with HCC development (OR = 0.95, 95%CI = 0.84–1.06) (Table 3, Figure 2B). When it came to HLA-DP rs9277535 and HLA-DQ rs7453920, consistent with all the studies [7], [8], [20], [21], the two polymorphisms (rs9277535: 3386 HCC patients and 5621 HBV patients; rs7453920: 2813 HCC patients and 3347 HBV patients) showed no connection with HCC development (allele model, rs9277535, OR = 1.04, 95%CI = 0.97–1.11; rs7453920, OR = 0.89, 95%CI = 0.76–1.02) (Table 3, Figure 2C, 2D). Two studies [14], [15] investigated STAT4 rs7574865 with HCC development, combined with our present data (in total, 962 HCC patients and 1418 HBV patients), we failed to find any significant association with HCC development (dominant model, OR = 0.87, 95%CI = 0.74–1.03, p = 0.113) (Table 3, Figure S1). Sensitivity analysis was performed through omitting studies with rather small sample size. And results were not significantly influenced (data now shown) and our conclusions were credible. Egger’s test showed that no publication bias existed among all the polymorphisms (Figure S2).

**Table 3:** Meta-analysis results of HLA-DP/DQ and STAT4 polymorphisms with HCC development.

SNPs	Gene	case/control	OR		95%CI	I^2^	Effects model
rs3077	HLA-DP	2997/3738		0.94	0.87–1.02	46.7%	F
rs3077	HLA-DP	2997/3738		0.95	0.84–1.06	46.7%	R
rs9277535	HLA-DP	3386/5621		1.04	0.97–1.11	5.7%	F
rs7453920	HLA-DQ	2813/3347		0.89	0.76–1.02	28.0%	F
rs7574865	STAT4	962/1418		0.87	0.74–1.03	0.0%	F

Note: OR, odds ratio; F, fixed-effects model; R, random-effects model.

### Haplotype analysis

Haplotype was constructed based on the 3 HLA-DP/DQ polymorphisms and were analyzed for their associations with HBV susceptibility, HBV seroclearance and HCC development. Based on rs3077 (minor allele A), rs9277535 (minor allele A) and rs7453920 (minor allele A), when comparing the target haplotype with remaining haplotype combinations, haplotype block GGG was significantly associated with HBV seroclearance (OR = 1.60, 95%CI = 1.31–1.96), block GAG favored virus clearance (OR = 0.56, 95%CI = 0.40–0.70). When comparison was made between HBV and HC subjects, haplotype block GGG still associated with increased risk of HBV infection (OR = 1.69, 95%CI = 1.34–2.14), while GAG and AAG connected with reduced risk of HBV infection (GAG, OR = 0.53, 95%CI = 0.37–0.77; AAG, OR = 0.70, 95%CI = 0.52–0.94). Moreover, we analyzed haplotype in HCC and HBV groups to acquire haplotype that was linked to HCC development. However, no haplotype presented significant association with HCC development (p>0.05) (Table 4).

**Table 4:** Haplotype analysis of rs3077, rs9277535 and rs7453920 with HBV susceptibility, seroclearance and HCC development.

Haplotype	case(freq)	control(freq)	OR	95%CI	p value
**(A)**					
AAA	24 (0.029)	20 (0.042)	0.66	0.36–1.22	0.183
AAG	132 (0.158)	97 (0.208)	0.7	0.52–0.94	0.016
AGG	71 (0.085)	37 (0.080)	1.05	0.69–1.59	0.811
GAG	67 (0.080)	65 (0.139)	0.53	0.37–0.77	0.0006
GGG	511 (0.661)	224 (0.481)	1.69	1.34–2.14	1.10E-05
**(B)**					
AAG	132 (0.158)	129 (0.178)	0.83	0.64–1.08	0.174
AGG	71 (0.085)	69 (0.096)	0.85	0.60–1.20	0.346
GAG	67 (0.080)	95 (0.131)	0.56	0.40–0.77	0.0004
GGA	25 (0.029)	22 (0.031)	0.92	0.51–1.64	0.769
GGG	511 (0.611)	348 (0.480)	1.6	1.31–1.96	5.91E-06
**(C)**					
AAG	77 (0.175)	132 (0.158)	1.1	0.81–1.50	0.55
AGG	28 (0.063)	71 (0.085)	0.71	0.45–1.12	0.136
GAG	37 (0.083)	67 (0.080)	1.02	0.67–1.55	0.942
GGG	280 (0.634)	511 (0.611)	1.04	0.81–1.34	0.732

(A) Haplotype analysis on HBV susceptibility; (B) Haplotype analysis on HBV seroclearance; (C) Haplotype analysis on HCC development.

## Discussion

In this study, we investigated HLA-DP/DQ and STAT4 polymorphisms with HBV susceptibility, viral clearance and HCC development in Han population residing in the Southwest of China. And our study first pointed out that currently hot spots (HLA-DP rs3077, rs9277535, HLA-DQ rs7453920, rs2856718) did not correlate with HCC development, either from experiment data or meta-analysis results, but they did associate with HBV susceptibility as well as HBV natural clearance. As for STAT4 rs7574865, it has no impact on HBV susceptibility and seroclearance, but its role in HCC development remains contentious.

Consistent with previous findings, HLA-DP rs3077, rs9277535, HLA-DQ rs7453920 still correlated with HBV seroclearance (p<0.05), and rs3077 allele A, rs9277535 allele A as well as rs7453920 allele A served as the protective role in HBV clearance. When analyzing HBV susceptibility between HBV patients and HC subjects, rs3077 and rs9277535 still associate with HBV susceptibility, but rs7453920 presented an insignificant result. This might be due to the limitation of sample size in HC group. Since the MAFs in HC and NC, HBV and HCC resemble, we divided all the patients into two groups: control (NC+SC) and HBV carrier (HBV+HCC). Next, we reappraised the correlation of rs7453920 with HBV susceptibility, and significant correlation existed. But the phenomenon implied that rs7453920 had weaker effect on HBV susceptibility compared with HLA-DP rs3077 and rs9277535.

As is listed before, only several studies [7], [8], [13]–[15], [20], [21] investigated the correlation of HLA-DP/DQ with HCC development, but quite conflicting results were acquired. In order to clarify this obscure correlation, we recruited HCC patients and replicated the experiments to further give an explanation on the conflicting results. But based on our experimental data and the haplotype analysis on HCC development, only negative results were achieved. Due to the limitation of our sample size, we subsequently performed meta-analysis to circumvent these confounding factors such as the sample size and the inherent heterogeneity in the study subjects. However, meta-analysis results again contradicted that HLA-DP rs3077, rs9277535 and HLA-DQ rs7453920 are not pertinent to HCC development. Besides, based on the weak or even insignificant connection of these SNPs with liver function indicators (Table S2), they would not be predictors for liver damage after HBV infection, and further implied that they could not predict the development of liver diseases, which, from another perspective, supported the conclusion both achieved from the experiment and meta-analysis. However, HLA-DQ rs2856718 seemed to significantly correlated with HCC development [7], and from meta-analysis, its role in HCC development seemed to be definite (OR = 0.88, 95%CI = 0.81–0.95) (Figure S3) and therefore we did not include it in this study.

Previous study has demonstrated that the risk alleles of HLA-DP polymorphisms (rs3077 G, rs9277535 G) associated with a decreased expression level of HLA-DP mRNA [22]. HLA-DP is expressed on the surface of antigen-presenting cells and belong to HLA-αmolecules, which can bind and present antigen epitopes to CD4+ T helper cell [23]. The decreased HLA-DP expression attributed to carrying risk alleles of HLA-DP rs3077 and rs9277535 might thus influence the antigen presentation, incurring immune evasion of the virus. One recent study [24] indicated HLA-DP polymorphisms might interact with HBV genome mutations to determine the outcomes of HBV infection, with HLA-DP protective genotypes relating to decreased prevalence of viral mutations which contributed to HCC development. Given the seemingly irrelevant role of HLA-DP/DQ in HCC development, the role of the association of HLA-DP/DQ polymorphisms with HBV viral mutations in HBV outcomes have dimmed. However in this study, we only validated those hot spots, which might lead to some missing causative polymorphisms. Considering the population heterogeneity, it would be better to sequence HLA-DP/DQ to unveil the polymorphisms that would play decisive role in HCC development.

STAT4 is a transcription factor which regulated immune response, transmitted signals from interleukin-12 (IL-12) and typeinterferon (IFN) to induce IFN-γ production [25]–[27]. Few studies had ever linked STAT4 with HBV diseases and HCC development before the GWAS studies came into being [12]. In light of current conflicting conclusions [12], [14], [15], we performed this validation study. Our present data doubted the discovery by the GWAS study that rs7574865 associated with HCC development [12]. Considering the limit of relatively small sample size, we performed this meta-analysis. Meta-analysis combined the two studies plus our current data including in total 962 HCC patients and 1418 HBV patients, and still failed to determine any significant association but only a trend with HCC development (OR = 0.87, 95% = 0.74–1.03). However, based on the sample size, the detection power of current meta-analysis still cannot challenge the conclusion drew by the GWAS study [12]. Therefore, in regard to STAT4 rs7574865 with HCC development, large cohort studies with high quality are warranted to validate this conclusion, particularly from multiple ethnicities to eliminate the race heterogeneity. Besides, we did not find any significant association of STAT4 rs7574865 with HBV seroclearance as well as the susceptibility when comparing HBV patients with seroclearance subjects and negative controls respectively. Accordingly, all these mentioned above suggested an irrelevant role of STAT4 rs7584865 in HBV susceptibility, at least in Chinese Han population, but seemingly a rather obscure association with HCC development awaiting more high-quality studies to corroborate.

Apart from what mentioned above, our study has several limitations. Firstly, we only replicated those currently hot SNPs but with conflicting relationship with HCC development, and the selected SNPs might not fully represent the related genes. In this way, we may have missed some representative and decisive gene polymorphisms. Secondly, when appraising the relationship of these gene polymorphisms with HCC development, we lacked virus genotype information, since Zhang et al. [24] suggested that different genotypes would have distinct outcomes stratified by HLA-DP polymorphism. Meanwhile, we lacked detailed information on the fibrosis stage and the presence of cirrhosis or not, so further studies are needed to comprehensively analyze the genotype frequencies of these gene polymorphisms in each stage of HBV related liver disease. Thirdly, the rather small number size limited the detection power in HCC group, though this has been remedied by the subsequent meta-analysis. And when analyzing the relationship of STAT4 rs7574865 with HCC using meta-analysis, we did not include the GWAS study, since all the other three studies held points similar to negative conclusion. If included, the positive relationship is definite, but this conclusion still warrants more studies in different ethnicities to substantiate. Moreover, since too few studies investigated each SNP and were included in the meta-analysis, we did not further conduct subgroup analysis stratified by quality of the studies, ethnicities etc., due to the probably insufficient statistical power. Fourthly, since the role of HLA-DP/DQ in HBV susceptibility as well as natural clearance is rather certain now, we did not take meta-analysis to summarize the associations.

These limitations notwithstanding, our study first summarized that current hot SNPs (rs3077, rs9277535, rs7453920) selected from HLA-DP/DQ did not associate with HCC development, from perspective of both experiment and meta-analysis. And consistent with previous studies, HLA-DP/DQ associated with HBV susceptibility and virus seroclearance. As for the recently emerged STAT4 rs7574865, experimental data contradicted its association with HBV susceptibility and HCC development. But given the rather limited sample size and the insignificant but tended-to-be significant meta-analysis results, the correlation of rs7574865 with HCC development still awaits large cohort studies to validate. Anyway, this study further corroborated the association of HLA-DP/DQ polymorphisms (rs3077, rs9277535 and rs7453920) with HBV seroclearance and first pinpointed their irrelevant role in HCC development. Future study could sequence HLA-DP/DQ genes to unveil the decisive polymorphisms associating with HCC development in multiple races and further explore how these polymorphisms impact on HBV clearance and HCC development and another study with large sample size to validate the correlation of rs7574865 with HCC development.

## Supporting Information

Figure S1 **Forest plot for association of STAT4 rs7574865 with HCC development.**
(TIF)

Figure S2 **Publication bias of the four polymorphisms discussed in this study.** (A) Publication bias for rs3077; (B) Publication bias for rs9277535; (C) Publication bias for rs7574865; (D) Publication bias for rs7453920.
(TIF)

Figure S3 **Forest plot for association of HLA-DQ rs2856718 with HCC development.**
(TIF)

Table S1 **Information of primers used in this study.**
(DOC)

Table S2 **Association of HLA-DP/DQ and STAT4 polymorphisms with clinical indicators.**
(DOC)

Table S3 **Clinical characteristics of the enrolled studies in the meta-analysis.**
(DOC)

Table S4 **Quality assessment for all included studies using the Newcastle-Ottawa Scale.**
(DOC)

Checklist S1 **Prisma checklist for the meta-analysis.**
(DOCX)
