# Supplementary figures and images for: Association of HLA-DP/DQ and STAT4 Polymorphisms with HBV Infection Outcomes and a Mini Meta-Analysis
Source: PLoS One. 2014 Nov 3;9(11):e111677. doi: 10.1371/journal.pone.0111677 (PMC4218798; doi:10.1371/journal.pone.0111677)

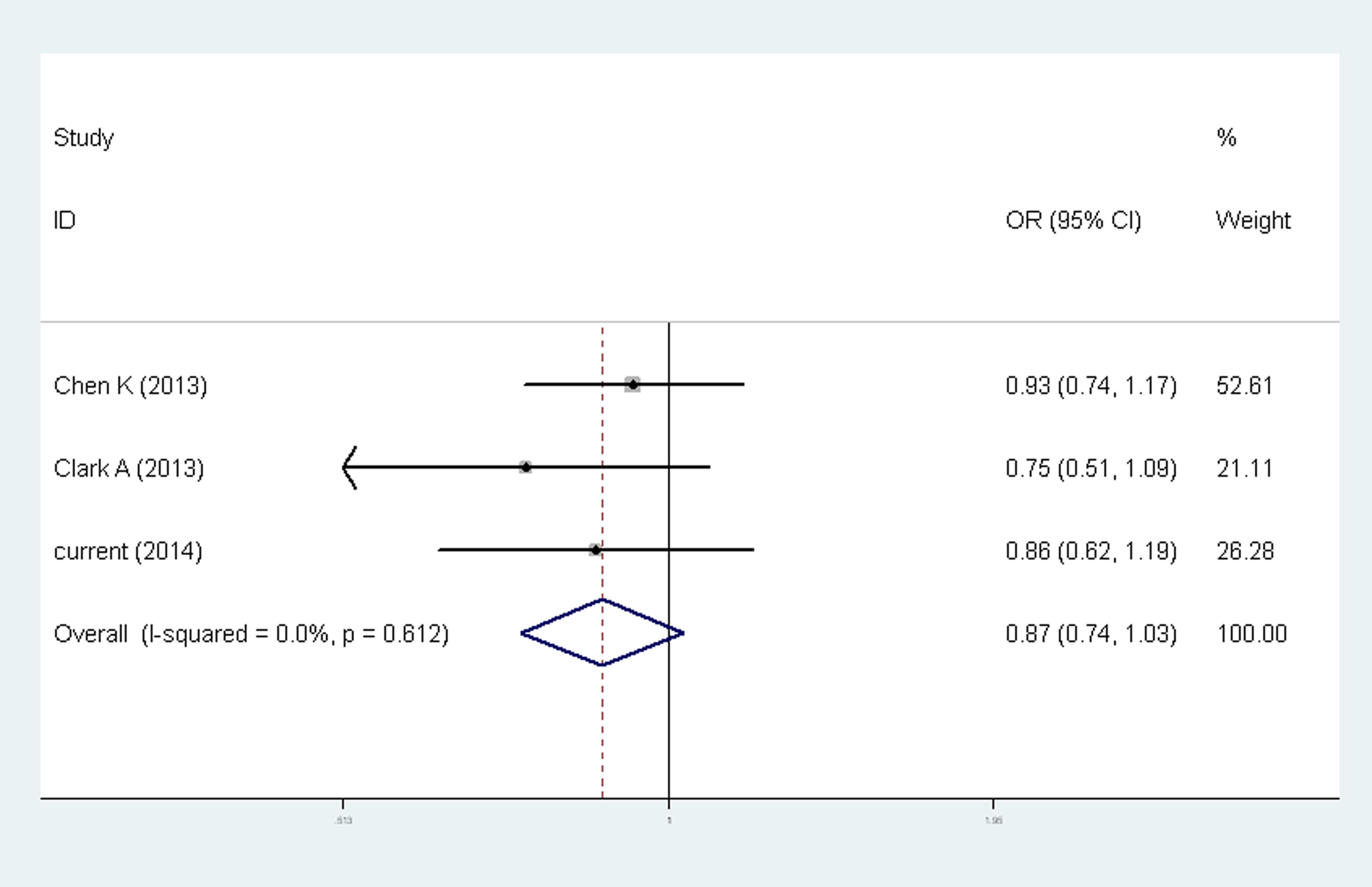

Supplement: Figure S1 — Forest plot for association of STAT4 rs7574865 with HCC development. (TIF) [file pone.0111677.s001.tif]

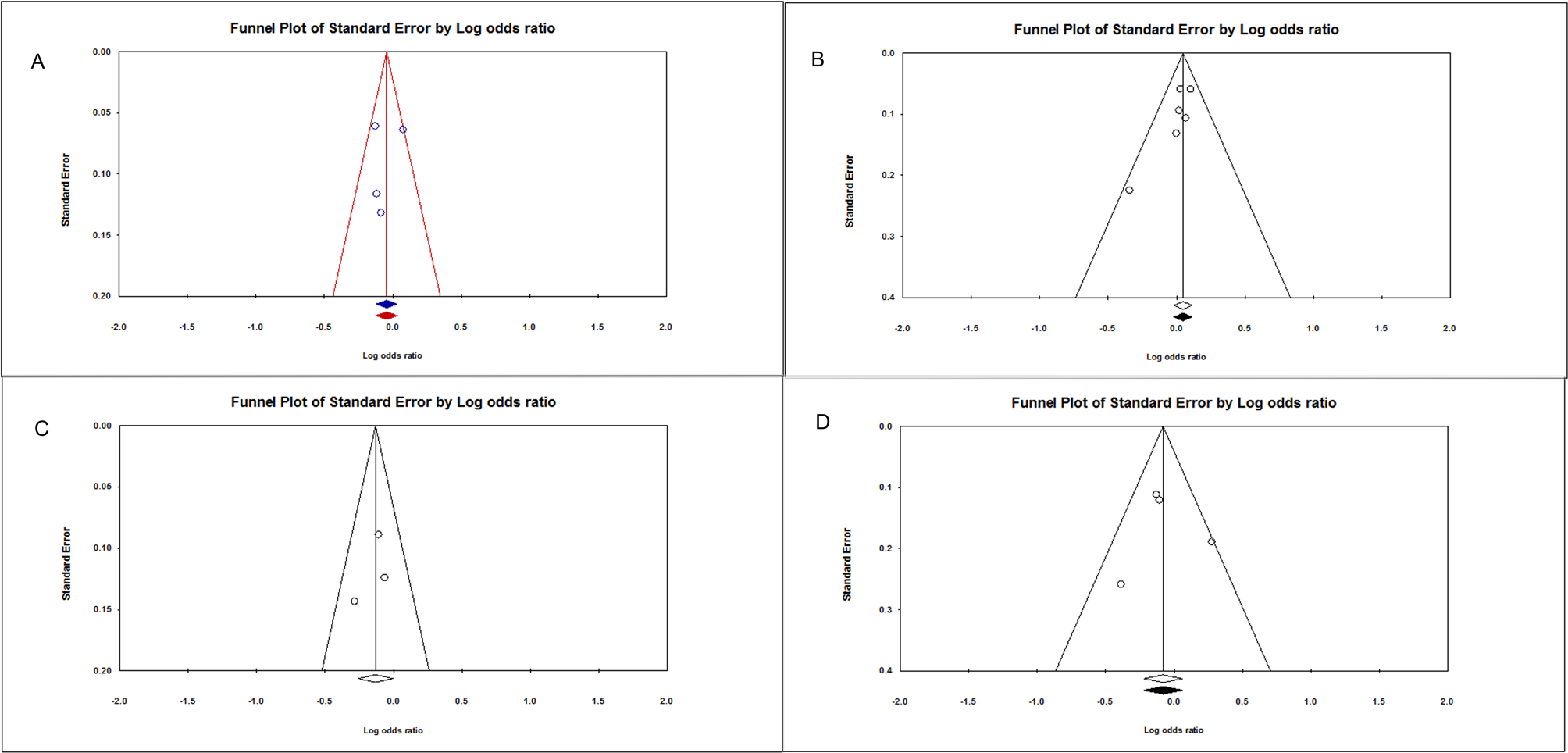

Supplement: Figure S2 — Publication bias of the four polymorphisms discussed in this study. (A) Publication bias for rs3077; (B) Publication bias for rs9277535; (C) Publication bias for rs7574865; (D) Publication bias for rs7453920. (TIF) [file pone.0111677.s002.tif]

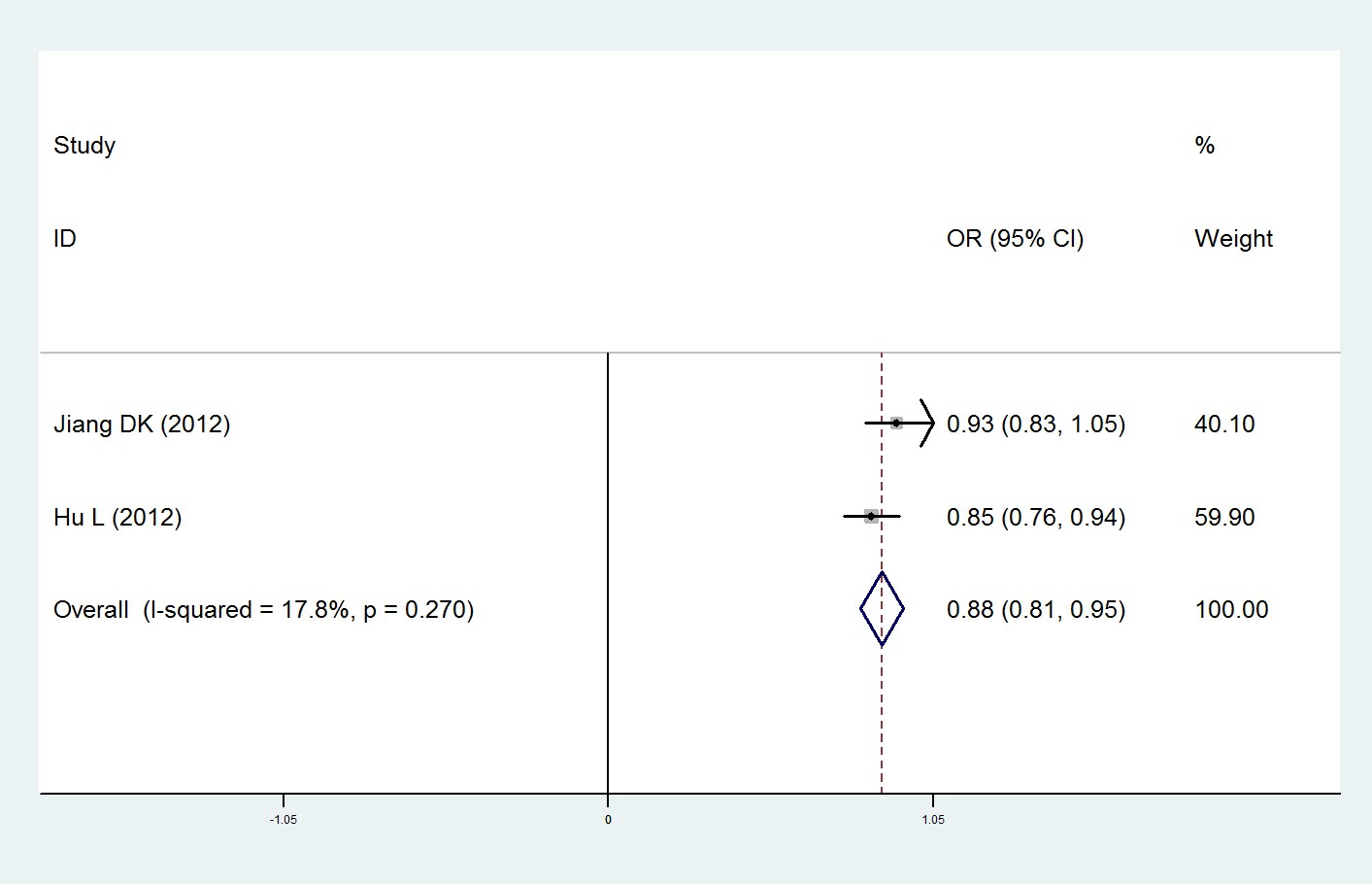

Supplement: Figure S3 — Forest plot for association of HLA-DQ rs2856718 with HCC development. (TIF) [file pone.0111677.s003.tif]
