# Supplementary material for: Association of HLA-DP/DQ and STAT4 Polymorphisms with HBV Infection Outcomes and a Mini Meta-Analysis
Source: PLoS One. 2014 Nov 3;9(11):e111677. doi: 10.1371/journal.pone.0111677 (PMC4218798; doi:10.1371/journal.pone.0111677)
Supplement: Table S1 — Information of primers used in this study. (DOC) [file pone.0111677.s004.doc]

**Table S1. Information of primers used in this study.**

| Polymorphism | Sequence(5’-3’) | |
| --- | --- | --- |
| rs3077 | Primer | F：TCAGCTTTTCTTCTCACTTCATGTG |
|  |  | R：GAGCTTGAAGGGTCAGCAATTC |
|  |  |  |
| rs9277535 | Primer | F：AATGGTGAGCAGACTGCAAATCT |
|  |  | R：TGGTAATGATAAAACATGCTCTCAGTAA |
|  |  |  |
| rs7453920 | Primer | F：TTTAGGGAGGTAAGAGGGAAAGC |
|  |  | R：CGAGAACGCCCTGATCTAAGA |
|  |  |  |
| rs7574865 | Primer | F: AAAGAAGTGGGATAAAAAGAAGTTTG |
|  |  | R: CCACTGAAATAAGATAACCACTGT |
