# Supplementary material for: Association of HLA-DP/DQ and STAT4 Polymorphisms with HBV Infection Outcomes and a Mini Meta-Analysis
Source: PLoS One. 2014 Nov 3;9(11):e111677. doi: 10.1371/journal.pone.0111677 (PMC4218798; doi:10.1371/journal.pone.0111677)
Supplement: Table S2 — Association of HLA-DP/DQ and STAT4 polymorphisms with clinical indicators. (DOC) [file pone.0111677.s005.doc]

**Table S2. Association of HLA-DP/DQ and STAT4 polymorphisms with clinical indicators**

| Group | rs3077 | | | | rs9277535 | | | | rs7574865 | | | |
| --- | --- | --- | --- | --- | --- | --- | --- | --- | --- | --- | --- | --- |
|  | GG | AG | AA | P | GG | AG | AA | P | GG | GT | TT | P |
| **NC** | 100 | 115 | 22 |  | 83 | 110 | 40 |  | 97 | 113 | 27 |  |
| ALT(IU/L) | 16 (11-22) | 16 (12-22) | 16.5 (12.75-24) | 0.796 | 16 (11-21) | 16 (12-23) | 16.5 (12-28.25) | 0.573 | 17 (12.5-24.5) | 16 (11.5-22) | 13 (10-20) | 0.19 |
| AST(IU/L) | 18.5 (15-23) | 19 (15-21) | 17.5 (15-24) | 0.852 | 19 (15-23) | 18 (15-21) | 19 (15-24) | 0.777 | 19 (16-22) | 19 (15-23) | 16 (15-19) | 0.154 |
| TB (μmol/L) | 10.5 (7-14) | 11 (9-13.7) | 10.5 (7.7-16.0) | 0.742 | 10.6 (7.7-14.7) | 11.7 (8.8-15.2) | 12.1 (8.0-14.9) | 0.507 | 11.6 (7.9-14.8) | 11.7 (8.9-15.0) | 10.5 (7.5-14.7) | 0.76 |
| TP (g/L) | 68.5 (65-73) | 70 (65-74) | 68.5 (66.7-73.2) | 0.48 | 67.8 (65.0-73.7) | 70.2 (66.4-74.1) | 68.7 (65.4-73.3) | 0.33 | 69.1 (65.8-73.9) | 69.1 (64.5-73.5) | 71.3 (65.3-75.9) | 0.353 |
| ALB (g/L) | 43 (41-45) | 44 (41-46.7) | 42.5 (40.7-47) | 0.241 | 43.5 (40.3-45.5) | 44.5 (41.9-47.0) | 43.6 (40.8-46.9) | 0.06 | 43.9 (41.6-46.5) | 44.1 (41.3-46.5) | 41.8 (38.1-46) | 0.223 |
| GGT(IU/L) | 15 (11.25-25) | 17 (12-26.7) | 16 (11.7-24) | 0.768 | 15 (11-26) | 15 (11-24.7) | 19 (14-35) | 0.282 | 18 (12-29) | 15 (11-24.7) | 13 (10-22) | 0.091 |
| PLT | 165 (115-207.5) | 165 (135.214) | 174 (131-226.2) | 0.729 | 175 (135-221) | 165 (118.5-201) | 159.5 (126-225) | 0.359 | 168 (126-222) | 159 (124-204) | 173 (147-190) | 0.466 |
| **SC** | 175 | 185 | 36 |  | 136 | 188 | 46 |  | 181 | 157 | 53 |  |
| ALT(IU/L) | 19 (14-25) | 20 (14-28) | 20.5 (15-27.5) | 0.45 | 20 (15-27.75) | 20 (14-26) | 18.5 (14-28.25) | 0.624 | 20 (14-27.25) | 20 (14-27) | 19 (15-24.5) | 0.838 |
| AST(IU/L) | 20 (17-24) | 21 (18-26) | 21.5 (18.2-24.7) | 0.072 | 21 (17-26) | 20 (17-24) | 21 (17-24) | 0.379 | 21 (18-26) | 20 (17-24) | 20 (17-25) | 0.132 |
| TB (μmol/L) | 11.4 (9-14.8) | 12 (9.7-15.8) | 12.6 (9.5-18.2) | 0.067 | 11.6 (9.3-15.4) | 12 (9.5-15.5) | 11.6 (9.7-16.1) | 0.869 | 11.8 (9.3-15.3) | 11.6 (9.5-15.6) | 11.5 (9.3-15.5) | 0.795 |
| TP (g/L) | 71.8 (67.8-75.3) | 71.1 (67.9-74.6) | 70.9 (68.4-73.1) | 0.654 | 71.4 (67.7-75.1) | 71.2 (67.9-74.4) | 70.6 (67.4-74.8) | 0.849 | 71.2 (68.1-75.5) | 71.1 (66.7-74.0) | 72.3 (68.5-75.5) | 0.171 |
| ALB (g/L) | 44.9 (42.6-47.4) | 45.7 (43-47.9) | 45.4 (43.8-48.0) | 0.298 | 45.2 (42.6-47.6) | 45.3 (43.2-47.6) | 45.2 (42.4-47.9) | 0.922 | 45.3 (43.2-47.5) | 45.3 (42.2-47.3) | 45.3 (43.8-48.1) | 0.323 |
| GGT(IU/L) | 17 (12-26.7) | 18 (12-31) | 20.5 (13-41.25) | 0.392 | 18 (13-29) | 18 (12-32) | 16.5 (11-24.7) | 0.527 | 17 (12-24) | 20 (13-33) | 16 (12.5-32) | 0.289 |
| PLT | 182 (144-217) | 178 (136-211) | 164 (126-206) | 0.229 | 181 (141-221) | 178 (141-207) | 165 (127-214) | 0.577 | 170 (137-208) | 183 (143-221) | 181 (142-205) | 0.176 |
| **HBV** | 233 | 171 | 33 |  | 226 | 179 | 31 |  | 190 | 204 | 46 |  |
| ALT(IU/L) | 25 (18-39) | 24 (17-36.5) | 26 (17-72) | 0.258 | 25 (18-38) | 24 (17-38) | 23 (17.25-44.75) | 0.895 | 25 (18-38) | 25 (17-40) | 25 (16.5-34.5) | 0.797 |
| AST(IU/L) | 26 (20-35) | 24 (20-30) | 28 (23-49) | 0.092 | 25 (20-32) | 24 (20-32) | 26 (21.5-35) | 0.469 | 25 (20.5-32.5) | 25 (20-34) | 25 (20.5-31.5) | 0.928 |
| TB (μmol/L) | 13.7 (10.1-17.6) | 14.3 (10.9-18.4) | 13.6 (9.7-18.9) | 0.518 | 13.6 (10.5-17.5) | 13.9 (10.3-18.4) | 15.4 (11.2-18.6) | 0.702 | 13.9 (10.8-18.1) | 13.4 (10.5-17.7) | 13.5 (9.6-17.5) | 0.61 |
| TP (g/L) | 71.7 (67.8-75.2) | 72.8 (67.2-76.6) | 70.4 (63.5-74.8) | 0.118 | 71.4 (67.4-75.5) | 72.7 (67.8-75.9) | 70.1 (64.7-75.1) | 0.138 | 71.8 (67.6-75.2) | 71.8 (67.7-75.2) | 70.2 (67.1-74.1) | 0.515 |
| ALB (g/L) | 44.6 (41.9-47.0) | 45.4 (42.5-47.5) | 44.8 (40.3-46.7) | 0.194 | 44.5 (41.8-47.2) | 45.4 (42.6-47.7) | 44.7 (40.7-47.1) | 0.143 | 45.1 (42.2-47.8) | 44.5 (41.9-47.1) | 44.5 (42.6-47.3) | 0.599 |
| GGT(IU/L) | 22 (14-37) | 20 (13-32) | 23 (13-47) | 0.287 | 22 (13-37.7) | 20 (13-33) | 19 (14-34) | 0.772 | 20 (13-36) | 22 (14-40) | 22 (12-31) | 0.417 |
| PLT | 149 (116-186) | 156 (116-198) | 148 (114-219) | 0.483 | 146 (114-185) | 156 (120-199) | 138 (91.7-165.5) | 0.149 | 152 (107-192) | 146 (116-189) | 167.5 (132-200) | 0.195 |
| **HCC** | 128 | 78 | 18 |  | 118 | 84 | 19 |  | 104 | 93 | 25 |  |
| ALT(IU/L) | 40.5 (26.2-64.7) | 38.5 (28-60.75) | 42.5 (25.7-75.0) | 0.788 | 42 (27-64.5) | 38 (26.5-59.5) | 41 (25.5-75) | 0.892 | 38.5 (28.7-59.25) | 43 (25-68) | 39 (25.5-124.5) | 0.854 |
| AST(IU/L) | 46 (32-69.75) | 37 (29-57.25) | 55 (34.5-78.25) | **0.05** | 45 (31.5-64.5) | 41 (29-66.75) | 51.5 (31.7-73.7) | 0.541 | 44 (32-62.5) | 45 (30.5-70) | 37 (28-86) | 0.82 |
| TB (μmol/L) | 15.9 (12.1-21.2) | 14.2 (10.6-18.7) | 20 (15-23.5) | 0.075 | 16.6 (12.2-21.3) | 13.8 (10.6-18.5) | 19.1 (12.0-22.7) | 0.114 | 15.2 (12.1-20.3) | 16.9 (12.2-21.1) | 13.5 (9.8-26.1) | 0.597 |
| TP (g/L) | 70.9 (65.9-73.9) | 70.6 (67.2-73.7) | 66.4 (63.4-69.7) | 0.124 | 71.3 (66.2-74.9) | 69.5 (65.8-73.3) | 68.5 (63.5-71.1) | 0.166 | 69.9 (66.3-73.4) | 71.3 (65.7-73.8) | 69.8 (64.6-74.6) | 0.966 |
| ALB (g/L) | 41.4 (37.7-44.2) | 40.9 (38.1-43.6) | 39.7 (36.5-43.5) | 0.711 | 40.9 (37.7-43.6) | 40.7 (37.1-43.8) | 42.6 (39.3-45.8) | 0.082 | 40.9 (37.7-43.6) | 40.7 (37.1-43.8) | 42.6 (39.3-45.8) | 0.082 |
| GGT(IU/L) | 81 (49-178) | 71 (39-124) | 92 (53-263) | 0.328 | 85.5 (44-189.5) | 66 (44-112) | 74 (43-202) | 0.436 | 78 (49-147.7) | 69 (44.5-172.5) | 77 (27.5-156.2) | 0.891 |
| PLT | 125 (88-167) | 105 (67-173) | 93 (62-143) | 0.227 | 116 (70-178) | 110 (81-167) | 104 (56.5-151) | 0.664 | 100 (71.5-162) | 124 (75-168) | 144 (101.5-186.7) | 0.205 |

Note: Data were shown as median with interquartile range and Kruskal-Wallis H test was used to analyze the difference among the three groups; NC, negative control; SC, spontaneous clearance subjects; HBV, hepatitis b virus carrier; HCC, hepatocellualr carcinoma; ALT, alanine aminotransferase; AST: aspartate aminotransferase; ALB, albumin; TB, total bilirubin; TP, total protein; GGT, gamma-glutamyl transpeptidase
