# Supplementary material for: Association of HLA-DP/DQ and STAT4 Polymorphisms with HBV Infection Outcomes and a Mini Meta-Analysis
Source: PLoS One. 2014 Nov 3;9(11):e111677. doi: 10.1371/journal.pone.0111677 (PMC4218798; doi:10.1371/journal.pone.0111677)
Supplement: Table S3 — Clinical characteristics of the enrolled studies in the meta-analysis. (DOC) [file pone.0111677.s006.doc]

**Table S3. Clinical characteristics of the enrolled studies in the meta-analysis**

| **Author** | **year** | **Ethnicity** | **Design** | **Detection method** | **No. of Case** | **No. of Control** | **Mean age(Case/Control)** | **Male(Case/Control)** |
| --- | --- | --- | --- | --- | --- | --- | --- | --- |
|
|
| An P[8] | 2011 | Han | Case-control | Taqman assay | 265 | 591 | 51.4/46.0 | 169/372 |
| Hu L[7] | 2012 | Han | Case-control | Taqman assay | 1344 | 1344 | 52.9/52.7 | 1104/1139 |
| Chen K[15] | 2013 | Han | Case-control | Taqman assay | 506 | 772 | 53.9/35.7 | 425/572 |
| Clark A[14] | 2013 | Vietnamese | Case-control | RtPCR+FRET | 239 | 206 | 52/40 | 208/159 |
| Li J[20] | 2011 | Han | Case-control | Taqman assay | 387 | 1874 | 49.8/38.4 | 342/1284 |
| Al-Qahtani AA[21] | 2014 | Saudi nationals | Case-control | PCR genotyping or Taqman | 81 | 200 | 51/41 | 71/497 |
| Jiang DK[12] | 2012 | Han | Case-control | GWAS | 1161 | 1353 | 51.6/48.4 | 898/917 |
