# Supplementary material for: Association of HLA-DP/DQ and STAT4 Polymorphisms with HBV Infection Outcomes and a Mini Meta-Analysis
Source: PLoS One. 2014 Nov 3;9(11):e111677. doi: 10.1371/journal.pone.0111677 (PMC4218798; doi:10.1371/journal.pone.0111677)
Supplement: Table S4 — Quality assessment for all included studies using the Newcastle-Ottawa Scale. (DOC) [file pone.0111677.s007.doc]

**Table S4. Quality assessment for all included studies using the Newcastle-Ottawa Scale**

| **Study** | **Year** | **Selection** | **Comparability** | **Exposure** | **Score** |
| --- | --- | --- | --- | --- | --- |
| An P | 2011 | ☆☆☆☆ | ☆ | ☆☆ | 7 |
| Hu L | 2012 | ☆☆☆☆ | ☆☆ | ☆☆ | 8 |
| Chen K | 2013 | ☆☆☆ | ☆ | ☆☆ | 6 |
| Clark A | 2013 | ☆☆☆ | ☆ | ☆☆ | 6 |
| Li J | 2011 | ☆☆☆ | ☆ | ☆☆ | 6 |
| Al-Qahtani AA | 2014 | ☆☆☆ | - | ☆☆ | 5 |
| Jiang DK | 2012 | ☆☆☆☆ | ☆ | ☆☆ | 7 |

Criteria for Selection: 1) Adequate definition of the cases; 2) Consecutive or obviously representative series of cases; 3) Representative controls; 4) No history of investigated diseases for control

Criteria for Comparability: 1) Study controls for age and sex; 2) Study controls for additional important factors, for example, smoking, alcohol, etc.

Criteria for Exposure: 1) Exposure ascertainment by blinded review or record; 2) Same method ascertaining exposures in both case and control; 3) Same non-response rate in both groups
